# The *ADH7* Promoter of *Saccharomyces cerevisiae* is Vanillin-Inducible and Enables mRNA Translation Under Severe Vanillin Stress

**DOI:** 10.3389/fmicb.2015.01390

**Published:** 2015-12-11

**Authors:** Trinh T. M. Nguyen, Aya Iwaki, Shingo Izawa

**Affiliations:** Laboratory of Microbial Technology, Graduate School of Science and Technology, Kyoto Institute of TechnologyKyoto, Japan

**Keywords:** *Saccharomyces cerevisae*, *ADH6*, *ADH7*, vanillin, translational repression, lignocellulosic biomass, bioethanol production

## Abstract

Vanillin is one of the major phenolic aldehyde compounds derived from lignocellulosic biomass and acts as a potent fermentation inhibitor to repress the growth and fermentative ability of yeast. Vanillin can be reduced to its less toxic form, vanillyl alcohol, by the yeast NADPH-dependent medium chain alcohol dehydrogenases, Adh6 and Adh7. However, there is little information available regarding the regulation of their gene expression upon severe vanillin stress, which has been shown to repress the bulk translation activity in yeast cells. Therefore, in this study, we investigated expression patterns of the *ADH6* and *ADH7* genes in the presence of high concentrations of vanillin. We found that although both genes were transcriptionally upregulated by vanillin stress, they showed different protein expression patterns in response to vanillin. Expression of Adh6 was constitutive and gradually decreased under vanillin stress, whereas expression of Adh7 was inducible, and, importantly, occurred under severe vanillin stress. The null mutants of *ADH6* or *ADH7* genes were hypersensitive to vanillin and reduced vanillin less efficiently than the wild type, confirming the importance of Adh6 and Adh7 in vanillin detoxification. Additionally, we demonstrate that the *ADH7* promoter is vanillin-inducible and enables effective protein synthesis even under severe vanillin stress, and it may be useful for the improvement of vanillin-tolerance and biofuel production efficiency via modification of yeast gene expression in the presence of high concentrations of vanillin.

## Introduction

Vanillin is generated as a by-product of the process of fermentable sugar production from lignocellulosic biomass. The vanillin concentration in the lignocellulosic hydrolysate can vary depending on the types of biomass materials and treatment methods, and a wide range of vanillin concentrations (1*–*26 mM) was reported in previous studies (Almeida et al., 2007; Heer and Sauer, 2008). Because vanillin is a potent inhibitor of fermentation, blocking the growth of yeast and subsequent fermentation in a dose-dependent manner, the toxicity of vanillin is one of the major barriers to reducing the production cost of bioethanol (Palmqvist and Hahn-Hägerdal, 2000a; Pampulha and Loureiro-Dias, 2000; Helle et al., 2003; Klinke et al., 2004). Therefore, breeding vanillin-tolerant yeast is an important prerequisite for efficient production of bioethanol from lignocellulosic biomass.

Vanillin was recently shown to repress initiation of translation and induce the formation of cytoplasmic messenger ribonucleoprotein (mRNP) granules such as processing bodies and stress granules in *Saccharomyces cerevisiae* (Iwaki et al., 2013b; Nguyen et al., 2014b), with high concentrations leading to limited translation of mRNAs and a reduction in overall protein synthesis levels. It is well known that glucose starvation also causes a rapid reduction in overall protein synthesis and induces the formation of mRNP granules (Ashe et al., 2000). Zid and O’Shea (2014) reported that small heat shock protein mRNAs such as *HSP26* and *HSP30* are efficiently translated during glucose starvation and showed that promoter sequences can influence not only the levels of mRNAs but also the efficiency of mRNA translation. There is currently no information regarding mRNAs that are efficiently translated during severe vanillin stress. To improve the vanillin tolerance of yeast cells, it would be useful to identify mRNAs that can be efficiently translated in the presence of severe vanillin stress.

Two NADPH-dependent enzymes, Adh6 and Adh7, have been shown to catalyze the reduction of vanillin to its less toxic form, vanillyl alcohol, *in vitro* (Larroy et al., 2002a,b). These two enzymes belong to the superfamily of medium-chain alcohol dehydrogenases, and they are the only representatives of the cinnamyl alcohol dehydrogenase family in *S*. *cerevisiae* (Larroy et al., 2002b). Their amino acid sequences show 64% identity and 80% similarity (Larroy et al., 2002b). The expression of *ADH6* and *ADH7* genes can be activated by the oxidative stress-responsive transcriptional factor Yap1 and the general stress-responsive transcriptional factor Msn2 (Berry and Gasch, 2008; Ma and Liu, 2010; Huebert et al., 2012; Nguyen et al., 2014a), and vanillin has been shown to activate Yap1 and Msn2 (Nguyen et al., 2014a,b). Because *adh7*Δ cells reduced low concentrations of vanillin (2.5 mM) more rapidly than did *adh6*Δ cells, Adh6 is considered to be the main enzyme responsible for reduction of vanillin *in vivo* (Iwaki et al., 2013b). However, currently, no information is available regarding the roles of Adh6 and Adh7 in the presence of high concentrations of vanillin.

In this study, we investigated the effect of severe vanillin stress on protein synthesis of Adh6 and Adh7. We found that only *ADH7* mRNA could be efficiently translated, although both *ADH6* and *ADH7* genes were transcriptionally upregulated under severe vanillin stress. The identification of genes that can be efficiently translated during severe vanillin stress would be useful to understand the mechanisms that could be applied to improve yeast tolerance and thus, improve biofuel production efficiency. We also examined the use of the *ADH7* promoter region to improve protein translation of unrelated proteins when cells are under severe vanillin stress and found that the *ADH7* promoter enabled protein synthesis of non-native genes such as *GPX2* and *GFP*. Our findings suggest that the *ADH7* promoter is useful for modification of yeast gene expression in the presence of high concentrations of vanillin.

## Materials and Methods

### Strains and Medium

*Saccharomyces cerevisiae* strain BY4742 (*MATα his3*Δ*1 ura3*Δ*0 leu2*Δ*0 lys2*Δ*0*) and its isogenic *adh6*Δ and *adh7*Δ null mutants (Open Biosystems Inc., Huntsville, AL, USA) were used in this study. Cells were cultured in 50 ml of SD medium (2% glucose, 0.67% yeast nitrogen base w/o amino acids, 20 mg/L uracil, 30 mg/L L-lysine HCl, 100 mg/L L-leucine, 20 mg/L L-histidine HCl) at 28°C with reciprocal shaking (120 rpm) in Erlenmeyer flasks (300 ml). Cell growth in the presence of vanillin was monitored by measuring optical density at 600 nm (OD_600_). Vanillin treatment was initiated from OD_600_ = 0.1 or 0.5 to examine its effect on the growth of knockout mutants or cells overexpressing *ADH6* gene driven by the *ADH7* promoter, respectively.

### Plasmids

The sequences of primers used in this study are listed in **Table 1**. Genomic DNA from BY4742 was used as a template to amplify yeast genes by PCR. pJK67 (Kahana et al., 1998) was used as a template to amplify the *GFP* gene by PCR.

**Table 1:** List of primers used in plasmid construction.

Name	Sequence
*ADH6_Orf_-*F	5′-CAATATCTAGATGCCGCTAGTCGTTGGTCA-3′
*ADH6_Orf_-*F2	5′-CGACAGAATTCCACATCCACATTCGAGG -3′
*ADH6_Orf_-*R	5′-CTTGACTCGAGAGTCTGAAAATTCTTTGTC-3′
*ADH7_Orf_-*F	5′-TTTCATCTAGACGTTGGTAATTGGGGTCCA-3′
*ADH7_Orf_-*R	5′-GTATACTCGAGATTTATGGAATTTCTTATC-3′
*ADH6_Pro_-*F	5′-AATAGGAGCTCCTCAACTCCATGGAGTGTT-3′
*ADH6_Pro_-*R	5′-TGTTGTCTAGAATTCCTTCCTCGAATGTGG-3′
*ADH7_Pro_-*F	5′-CCATGGAGCTCCACTAGATTGAGACCAGCC-3′
*ADH7_Pro_-*R	5′-AGCATTCTAGATTTGTATTTTTCAGTGGTT-3′
*ADH6_Ter_-*F	5′-GC AAGCTCGAGCTGACT AC AAGGATGACGATGAC AA GTAGGTTGTCAAGCTCTTGATAAATG-3′
*ADH6_Ter_-*R	5′-GAAAAGGTACCCAGATCTACCACCAAACCT-3′
*ADH7_Ter_-*F	5′-TAAATCTCGAGCTGACTAC AAGGATGACGATGAC AAG TAGTCTATATACGTAATATTTTTCAGAA-3′
*ADH7_Ter_-*R	5′-TTAACACGGTACCTTTCGGACGAAAATTGC -3′
*GFP-*F	5′-CCAACGAATTCCCGAGCTATGGCTAGCAAA-3′
*GFP-*R	5-TTCCTCTCGAGCTTTGTTAGCAGCCGGATC-3′
*GPX2-*F	5′-AACTTGAATTCCCCTGCATGGCTGACCTTG-3′
*GPX2-*R	5′-ACAAACTCGAGATTTACTTAACAGGCTTTG-3′


#### YIp*-ADH6-GFP*

A 0.8-kbp fragment encoding part of the open reading frame (ORF) of *ADH6* was amplified using primers *ADH6*_Orf_-F and *ADH6*_Orf_-R. The amplicon was digested with *Xba*I/*Xho*I and cloned into the *Xba*I/*Xho*I sites of pJK67 to construct YIp-*ADH6-GFP*.

#### YIp*-ADH6-FLAG*

The integrate-type plasmid YIp-*ADH6-FLAG* was constructed to estimate the protein levels of Adh6. This plasmid contained a *FLAG* tag sequence (encoded by 24 nt) immediately upstream of the stop codon and 3′-flanking region of *ADH6*. A 0.5-kbp fragment encoding a *FLAG* tag sequence, stop codon, and the 3′-flanking region of *ADH6* was amplified using primers *ADH6*_Ter_-F and *ADH6*_Ter_-R. The amplicon was digested with *Xho*I/*Kpn*I and cloned into the *Xho*I/*Kpn*I sites of YIp-*ADH6*-*GFP* to construct YIp-*ADH6*-*FLAG*. To integrate the *ADH6*-*FLAG* gene at the chromosomal *ADH6* locus, YIp-*ADH6*-*FLAG* was linearized by digesting with *Bst*XI and was then introduced into yeast cells.

#### YIp*-ADH7-GFP* and YIp*-ADH7-FLAG*

A 1.0-kbp fragment encoding part of the ORF of *ADH7* was amplified using primers *ADH7*_Orf_-F and *ADH7*_Orf_-R. The amplicon was digested with *Xba*I/*Xho*I and cloned into the *Xba*I/*Xho*I sites of pJK67 to construct YIp-*ADH7*-*GFP*. The integrate-type plasmid YIp-*ADH7*-*FLAG* was constructed to estimate the protein levels of Adh7. A 0.5-kbp fragment encoding a FLAG tag sequence, stop codon, and the 3′-flanking region of *ADH7* was amplified using primers *ADH7*_Ter_-F and *ADH7*_Ter_-R. The amplicon was digested with *Xho*I/*Kpn*I, and cloned into the *Xho*I/*Kpn*I sites of YIp-*ADH7*-*GFP* to construct YIp-*ADH7*-*FLAG*. To integrate the *ADH7*-*FLAG* gene at the chromosomal *ADH7* locus, YIp-*ADH7*-*FLAG* was linearized by digesting with *Bst*EII and was then introduced into yeast cells.

#### pRS316-*ADH*_Pro/Ter_

The promoter region (1.0 kbp) and the terminator region, including FLAG tag (0.5 kbp), of *ADH* genes were amplified using primer sets *ADH*_Pro_-F/R and *ADH*_Ter_-F/R, and they were cloned into the *Sac*I/*Xba*I and *Xho*I/*Kpn*I sites of pRS316 (Sikorski and Hieter, 1989), respectively, to construct pRS316-*ADH6*_Pro/Ter_, pRS316-*ADH7*_Pro/Ter_, and pRS316-*ADH7*_Pro_-*ADH6*_Ter_. The ORFs of the *GPX2* and *GFP* genes were amplified using the primer sets *GPX2*-F/R, and *GFP*-F/R, respectively, and cloned into the *Eco*RI/*Xho*I sites of pRS316-*ADH7*_Pro/Ter_ to construct pRS316-*ADH7*_Pro_-*GPX2*-*ADH7*_Ter_, and pRS316-*ADH7*_Pro_-*GFP*-*ADH7*_Ter_. Other pRS316 plasmid series were constructed in the same way.

#### pRS423-*ADH7*_Pro_-*ADH6*-*ADH7*_Ter_

The ORF of the *ADH6* gene was amplified using primers *ADH6*_Orf_-F2 and *ADH6*_Orf_-R and inserted into the *Eco*RI and *Xho*I sites of pRS316-*ADH7*_Pro/Ter_ to construct pRS316-*ADH7*_Pro_-*ADH6-ADH7*_Ter_. The *Sac*I and *Kpn*I region (2.5 kbp) of pRS316-*ADH7*_Pro_-*ADH6-ADH7*_Ter_ containing the *ADH7*_Pro_-*ADH6*-*ADH7*_Ter_ was isolated and cloned into pRS423 (Sikorski and Hieter, 1989) to construct pRS423-*ADH7*_Pro_-*ADH6-ADH7*_Ter_.

### Chemicals and Analysis Methods

Vanillin and dimethyl sulfoxide (DMSO) were obtained from Wako Pure Chemical Industries (Osaka, Japan). Stock solutions of 2 M vanillin were prepared in DMSO and stored at –30°C. Exponentially growing cells were harvested at OD_600_ = 0.5 and treated with varying concentrations of vanillin. Polysome profile analysis was performed using the method described by Inada and Aiba (2005). Polysome ratio was determined as the percentage of the area under polysomal ribosome peaks relative to the area under total ribosome peaks, according to the method of Hofmann et al. (2012). A Leica AF6500 (Leica Microsystems, Wetzlar, Germany) fluorescence microscopic system was used for analysis. Cells treated with vanillin were observed immediately after treatment without fixation. The concentrations of vanillin in the culture medium were measured by high performance liquid chromatography (HPLC) as described in a previous report (Nguyen et al., 2014b).

### Quantitative Real Time-PCR

The relative mRNA levels of the *ADH6* and *ADH7* genes were determined by quantitative real time-polymerase chain reaction (qRT-PCR). Total RNA was extracted by the method of Schmitt et al. (1990). The amount of total RNA extracted from each sample was approximately 100*–*150 μg, corresponding to 50 mL of cell culture. cDNA was synthesized using ReverTra Ace qPCR RT Master Mix FSQ-201 (Toyobo, Osaka, Japan), according to the manufacturer’s instructions. Analysis was performed using the sequence detection system (Thermal Cycler Dice Real Time System Lite, Takara Bio, Shiga, Japan) and SYBR^®^Premix Ex Taq^TM^II (Takara Bio, Shiga, Japan). The oligonucleotide sequences of the primers used for qRT-PCR were verified in previous studies (Liu et al., 2009) and are listed in **Table 2**. The results of several independent qRT-PCR experiments showed that the fluorescent signal intensity of the *ACT1* gene was not significantly changed after vanillin treatment, indicating that the mRNA levels of *ACT1* gene were almost the same at the different vanillin concentrations.

**Table 2:** List of primer pairs used in quantitative real time-PCR.

Gene	Orientation	Primer sequence
*ADH6*	Forward	5′-GAGAGCCTTAGCGTATTTCG-3′
*ADH6*	Reverse	5′-CCTGTGGTATCTGCGGATCT-3′
*ADH7*	Forward	5′-ATTTCCAACGCAAAGGATTG-3′
*ADH7*	Reverse	5′-CCTGTGGTATCTGCGGATCT-3′
*ACT1*	Forward	5′-TTGGATTCCGGTGATGGTGTTACT-3′
*ACT1*	Reverse	5′-TGAAGAAGATTGAGCAGCGGTTTG-3′


### Western Blot Analysis

After treatment of cells with vanillin in SD medium, a cell-free extract (CFE) in 100 mM potassium phosphate buffer (pH 7.4) was immediately prepared. The total protein concentrations of CFE were measured using the Protein Assay CBB Solution kit (Nacalai Tesque, Kyoto, Japan). Thirty micrograms of total protein was applied to each lane of a 12.5% polyacrylamide gel for SDS-PAGE analysis. Levels of FLAG-tagged proteins (Adh6, Adh7, and Gpx2) were monitored by western blotting using a monoclonal anti-FLAG M2 antibody (Sigma-Aldrich, St. Louis, MO, USA). Levels of GFP were monitored with an anti-GFP antibody (mFX75; Wako Pure Chemical Industries, Osaka, Japan). Pgk1 was used as a loading control, and its levels were monitored with a monoclonal anti-PGK antibody (A-6457; Molecular Probes, Eugene, OR, USA). The bands of western blot were quantified using ImageJ software (http://imagej.nih.gov/ij/).

## Results

### Differences in Protein Synthesis of Adh6 and Adh7 Under Severe Vanillin Stress

**FIGURE 1:**
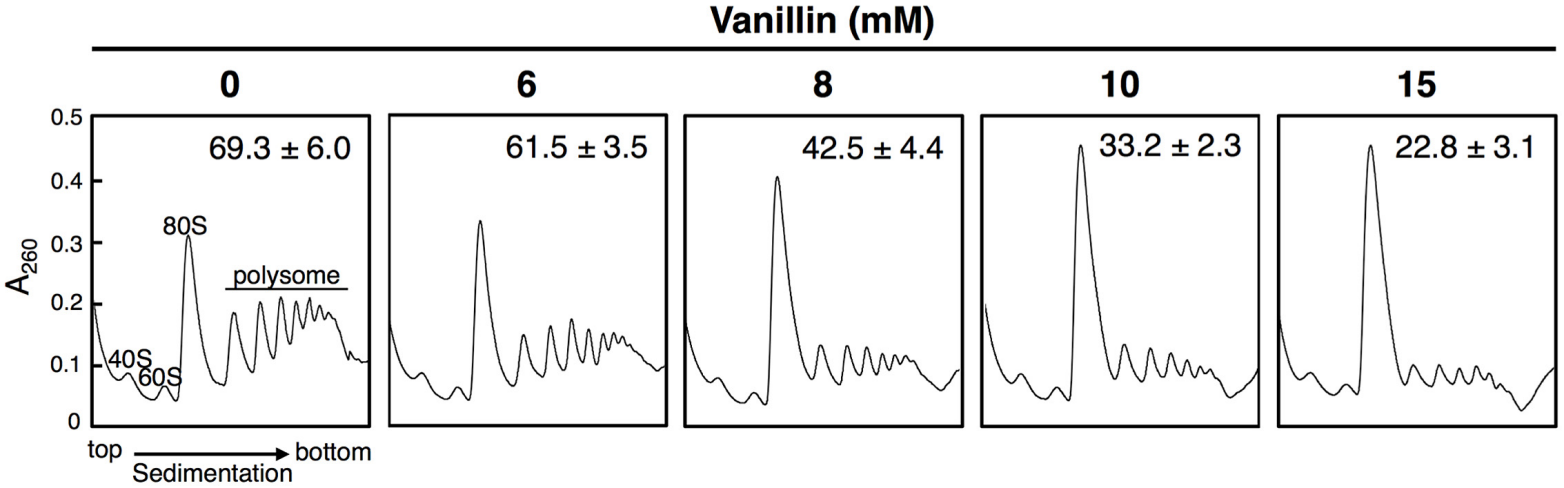
**High concentrations of vanillin inhibit translation initiation in *Saccharomyces cerevisiae* BY4742.** Cells in an exponential phase of growth in SD medium were treated with vanillin (6–15 mM) for 30 min. The polysome profile of cells treated with vanillin was determined. The polysome, 40S (small ribosomal subunit), 60S (large ribosomal subunit), and 80S (monosome) peaks are labeled. The numbers in the panels indicate the percentages of polysomal ribosomes. Data are represented as the mean ± SD of three independent experiments.

We previously reported that concentrations of vanillin greater than 7.5 mM severely repress protein translation in BY4741 and YPH250 yeast strains (Iwaki et al., 2013b; Nguyen et al., 2014b). We first verified that vanillin represses translation in the BY4742 strain used in this study by polysome profile analysis (**Figure 1**). A pronounced reduction in the polysome fraction was caused in BY4742 cells following treatment with 6*–*15 mM vanillin for 30 min, confirming that overall protein synthesis levels was markedly reduced by severe vanillin stress (i.e., using concentrations greater than 8 mM).

**FIGURE 2:**
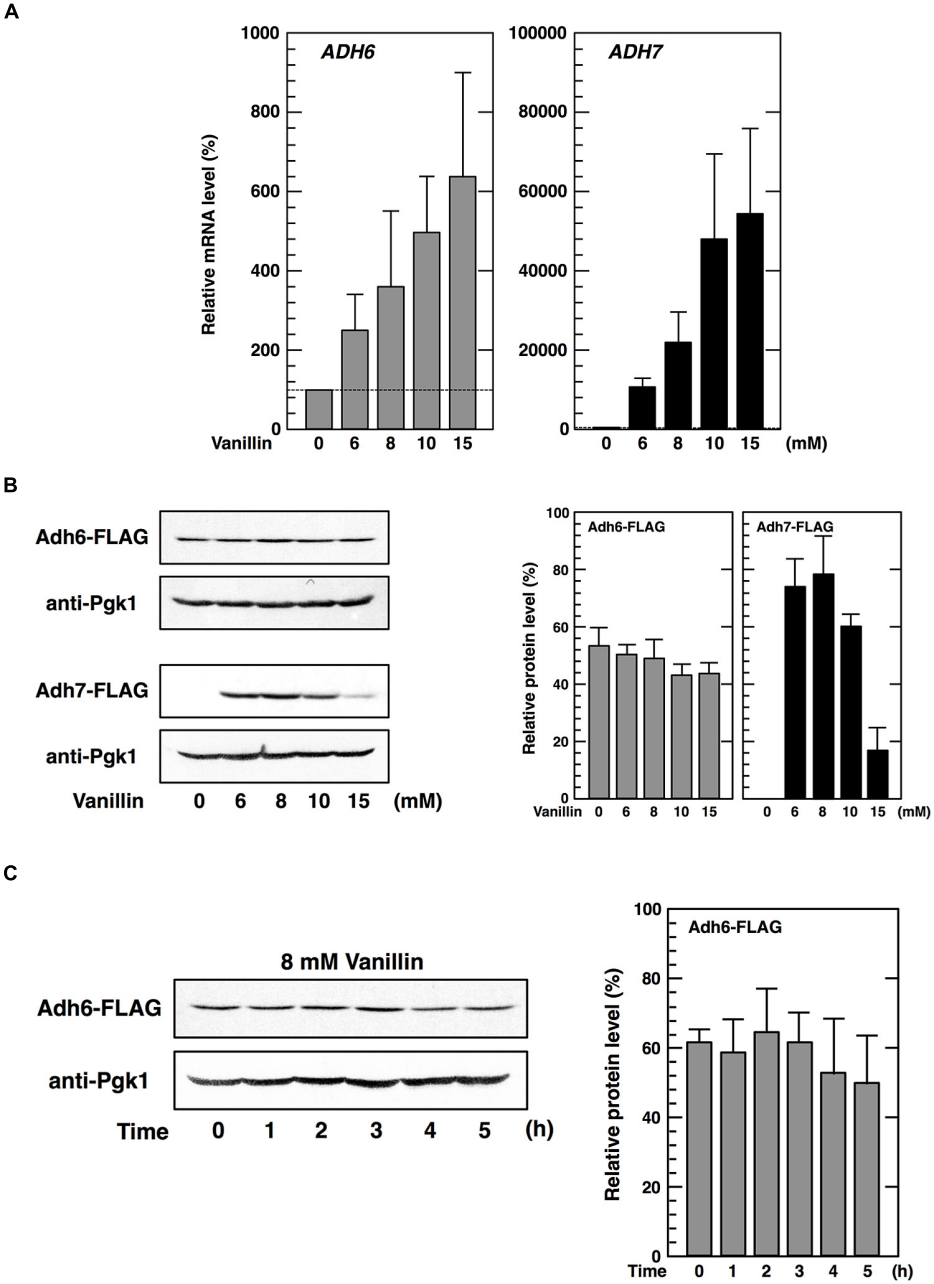
**Expression of the *ADH6* and *ADH7* genes under vanillin stress.** **(A)** The mRNA levels of *ADH6* and *ADH7* were analyzed by qRT-PCR. Cells in an exponential phase of growth were treated with vanillin (6–15 mM) for 60 min. To compare mRNA expression levels, the mRNA level of each gene was normalized to that of *ACT1*. The mRNA level in cells without vanillin treatment was considered 100%. Data are shown as mean ± SD of three independent experiments. **(B,C)** Levels of Adh6 and Adh7 protein expression were determined by western blot analysis using an anti-FLAG antibody. Cells carrying a FLAG-tagged chromosomal copy of *ADH6* or *ADH7* gene were treated with the indicated concentrations of vanillin for 60 min **(B)**, or treated with 8 mM vanillin for the indicated periods of time **(C)**. Pgk1 was used as a loading control. Protein levels of Adh6 and Adh7 were normalized to that of Pgk1 using ImageJ, and the intensity of Pgk1 band of each lane was considered 100%. Data are shown as the mean ± SD of three independent experiments.

Next, we investigated the effects of vanillin on the transcription of the *ADH6* and *ADH7* genes by qRT-PCR. As shown in **Figure 2A**, mRNA levels of both genes were significantly increased following treatment with 6*–*15 mM vanillin, indicating that both the *ADH6* and *ADH7* genes were transcriptionally upregulated by severe vanillin stress.

We then examined whether protein synthesis of Adh6 and Adh7 was also upregulated upon severe vanillin stress. Protein levels of Adh6 were reduced slightly in the presence of varying concentrations of vanillin for 60 min (**Figure 2B**). Furthermore, prolonged treatment (5 h) using 8 mM vanillin did not increase Adh6 protein synthesis (**Figure 2C**). Similar to that observed with Adh6, protein levels of Gpx2 (glutathione peroxidase) and Trx2 (cytoplasmic thioredoxin) did not increase upon severe vanillin stress (data not shown), although their mRNA levels were increased by exposure to vanillin (Nguyen et al., 2014a,b).

In contrast, levels of Adh7 protein synthesis were significantly altered upon vanillin stress. Adh7 protein was expressed at levels below detection limits under non-stressed conditions, but clear bands representing Adh7 were detected following vanillin treatment (**Figure 2B**). Protein synthesis of Adh7 was maximally induced by concentrations in the range of 8 mM vanillin, and was still detected in the presence of 15 mM vanillin, which was previously shown to severely repress bulk translation activity (**Figure 1**). These results suggest that the expression of the *ADH7* gene is vanillin-inducible and that *ADH7* mRNA can be efficiently translated even under severe vanillin stress, despite the repression of bulk translational activity.

### Expression of the *ADH7* Gene was Not Induced by the Deficiency of Adh6

**FIGURE 3:**
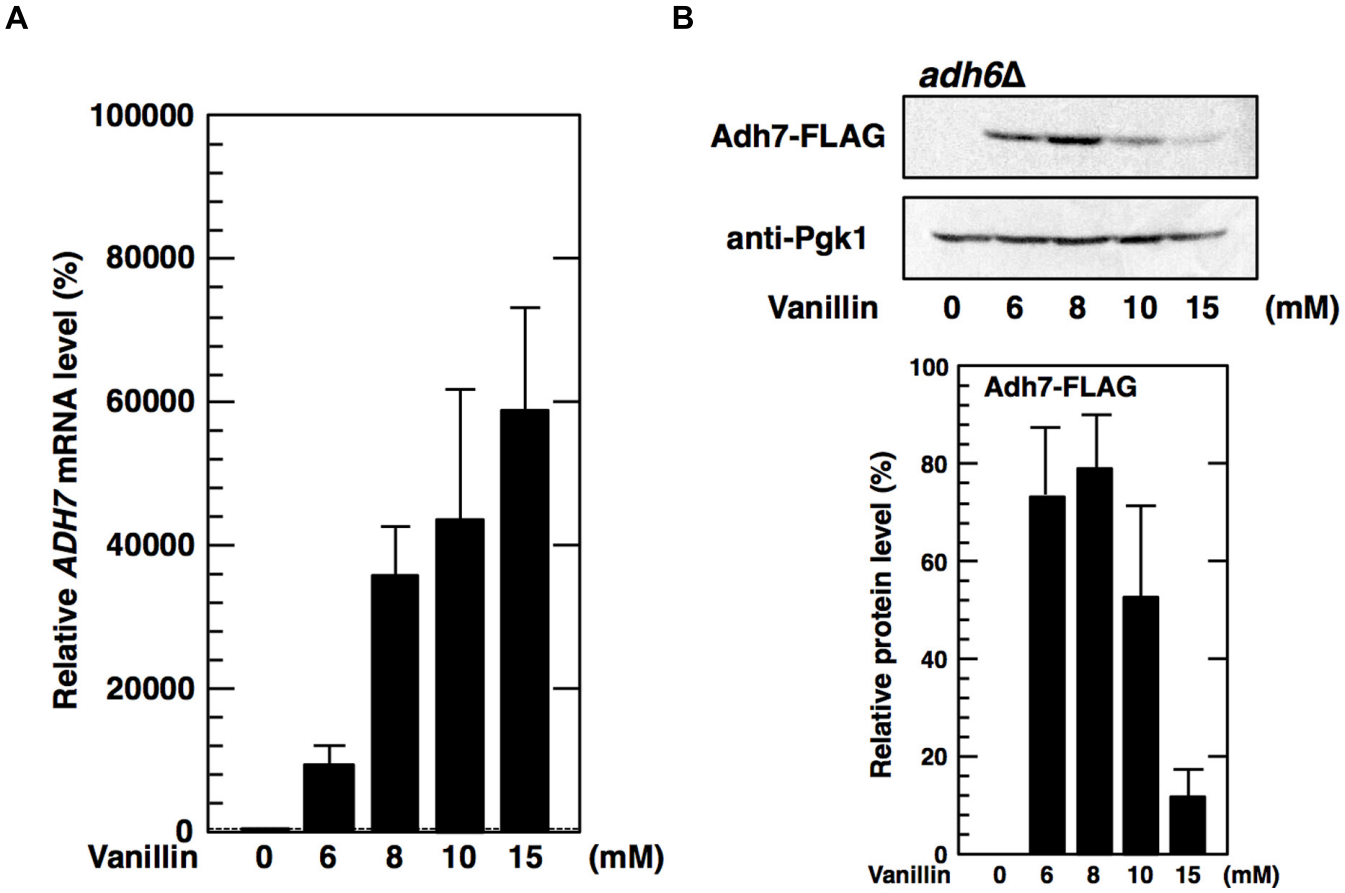
**Deletion of the *ADH6* gene has no significant effect on the expression of the *ADH7* gene.** The *adh6*Δ cells carrying a FLAG-tagged chromosomal copy of the *ADH7* gene were treated with vanillin for 60 min. **(A)** The mRNA levels of *ADH7* were analyzed using qRT-PCR. To compare mRNA expression levels, the mRNA level of each gene was normalized to that of *ACT1*. The mRNA level in cells without vanillin treatment was considered 100%. Data are shown as the mean ± SD of three independent experiments. **(B)** Adh7 levels were determined by western blot analysis using an anti-FLAG antibody. Pgk1 was used as a loading control. Protein levels of Adh7 were normalized to that of Pgk1 using ImageJ, the intensity of Pgk1 band of each lane was considered 100%. Data are shown as the mean ± SD of three independent experiments.

Because protein levels of Adh6 were not upregulated by vanillin, we considered whether a lack of Adh6 might be compensated for by the induction of *ADH7* expression. Therefore, we examined whether deletion of the *ADH6* gene affected the expression of the *ADH7* gene. However, there was no significant difference observed in either the basal or the inducible expression levels of *ADH7* mRNA or protein between the *adh6*Δ cells and the wild-type cells, and a similar pattern of response to vanillin was observed (**Figures 2A,B** and **3A,B**).

### Hypersensitivity of *adh6*Δ and *adh7*Δ Mutants to Vanillin

**FIGURE 4:**
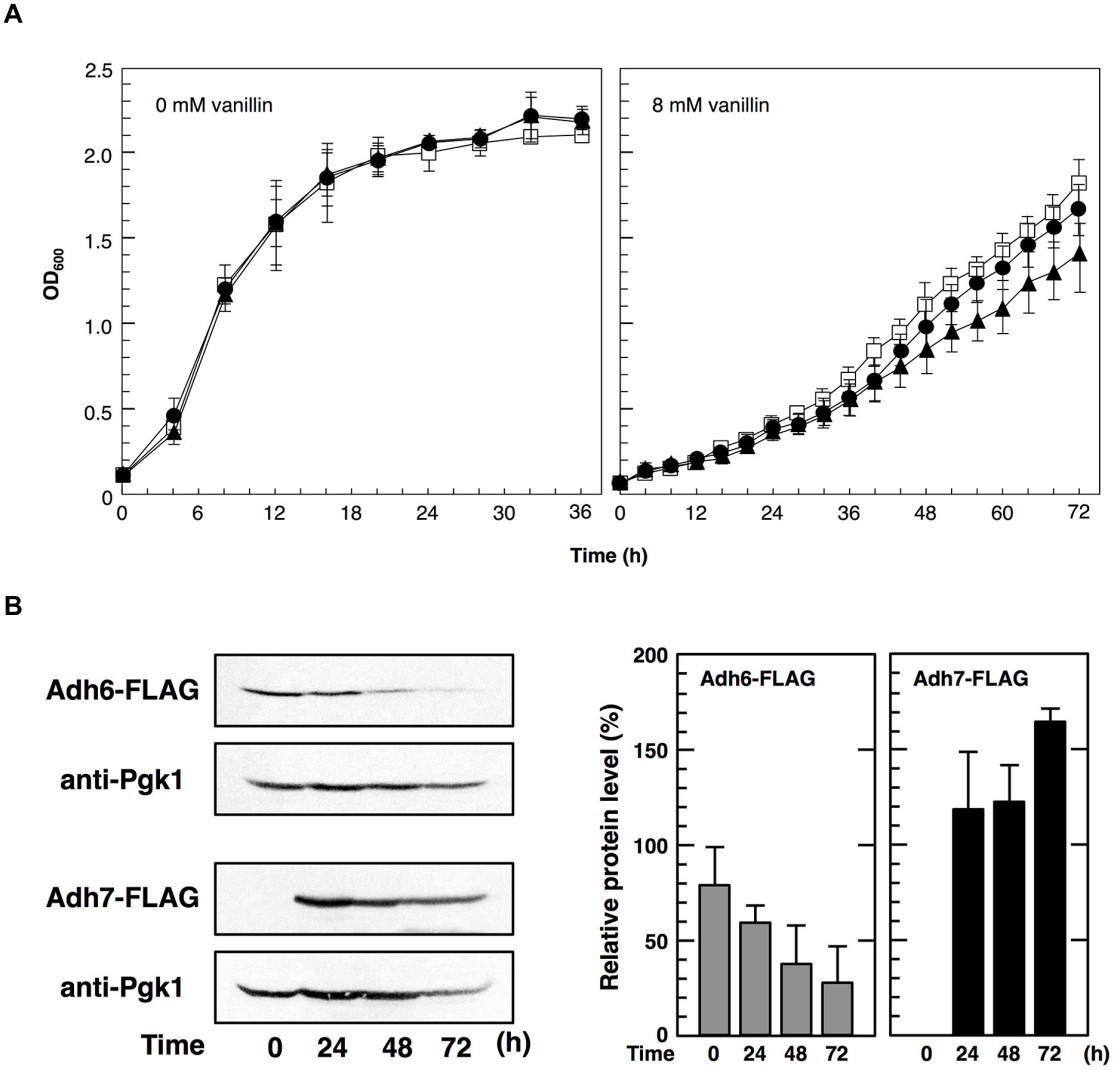
**Cells were cultured at 28°C in SD medium containing 8 mM vanillin.** **(A)** Growth of *adh6*Δ and *adh7*Δ cells was monitored by measuring the optical density at 600 nm (OD_600_). Data are shown as the mean ± SD of three independent experiments. The strains examined were as follows: open squares, wild type; closed circles, *adh6*Δ; closed triangles, *adh7*Δ. **(B)** Changes in Adh6 and Adh7 levels during cultivation were monitored by western blot analysis using an anti-FLAG antibody. Pgk1 was used as a loading control. Protein levels of Adh6 and Adh7 were normalized to that of Pgk1 using ImageJ, and the intensity of Pgk1 band of each lane was considered 100%. Data are shown as the mean ± SD of three independent experiments.

To examine the importance of Adh6 and Adh7 in tolerance to severe vanillin stress, we examined the growth of the null mutants (*adh6*Δ and *adh7*Δ) in the presence of vanillin (**Figure 4A**). Although the growth rate of both mutants was almost identical to that of the wild-type strain under non-stressed conditions, the presence of vanillin resulted in a slower growth rate of the null mutants compared with that of the wild-type strain. The growth rate of the *adh7*Δ cells was slower than that of the *adh6*Δ cells in the presence of 8 mM vanillin, suggesting that the inducible expression of *ADH7* is important for yeast tolerance against high concentrations of vanillin.

In order to further compare the importance of Adh6 and Adh7 in vanillin detoxification, we examined changes in vanillin concentration in the culture medium using HPLC (**Table 3**). The wild-type cells reduced 8 mM vanillin to almost zero after cultivation for 48 h. However, the mutant cells reduced vanillin less effectively than the wild-type cells, and the *adh7*Δ cells showed a slower rate of vanillin reduction than the *adh6*Δ cells. Additionally, the expression of *ADH7* gene was highly induced by 8 mM vanillin treatment and persisted till the end of the experiment, whereas the level of Adh6 protein gradually decreased during vanillin treatment for 72 h (**Figure 4B**). These results clearly indicate that Adh7 plays an important role under severe and long-term vanillin stress due to its high and stable expression under these conditions.

**Table 3:** Changes in vanillin concentrations (mM) in the culture medium during cultivation for 72 h.

Strain	Cultivation time (*h*)			
	
	0	24	48	72
Wild-type	8.00	4.58 ± 0.24	0.52 ± 0.12	N.D.
*adh6Δ*	8.00	4.76 ± 0.16	1.81 ± 0.26	0.02 ± 0.40
*adh7Δ*	8.00	5.59 ± 0.70	3.00 ± 0.29	0.89 ± 0.33


*Vanillin concentrations (mM) are presented as mean ± SD of three independent experiments. N.D., non-detectable.*

### The *ADH7* Promoter Enables Protein Synthesis under Severe Vanillin Stress

**FIGURE 5:**
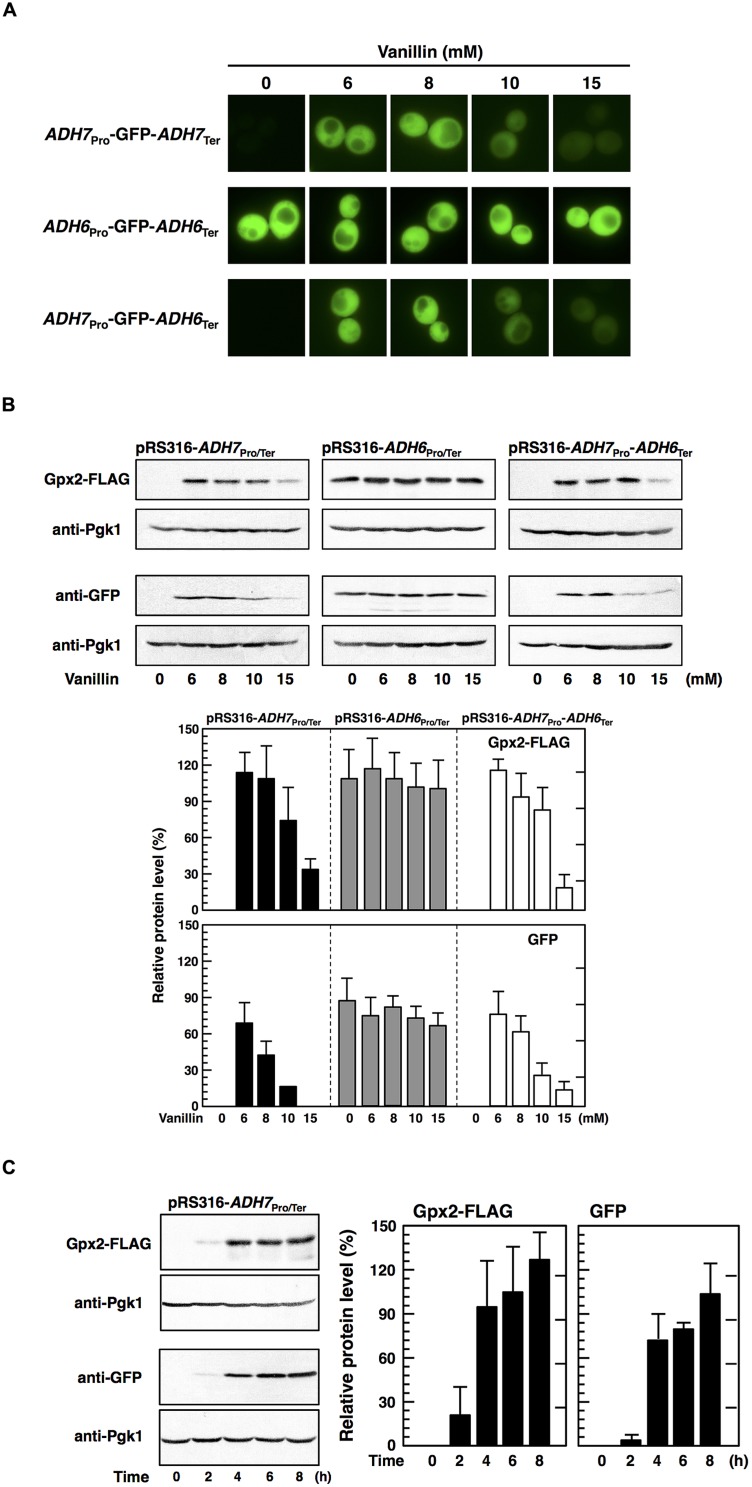
**The *ADH7* promoter region enables protein synthesis of non-native genes in the presence of high concentrations of vanillin.** Cells carrying the pRS316 plasmid series in an exponential phase of growth were treated with vanillin for 60 min **(A,B)**. **(A)** Fluorescence microscopic analysis was carried out to monitor the synthesis of GFP. **(B)** Protein levels of Gpx2 and GFP were determined by western blot analysis. **(C)** Expression of the *GPX2* and *GFP* genes during the treatment with 15 mM vanillin for 8 h was examined. Protein levels of Gpx2 and GFP were normalized to that of Pgk1 using ImageJ, and the intensity of Pgk1 band of each lane was considered 100%. Data are shown as the mean ± SD of three independent experiments.

Zid and O’Shea (2014) recently reported that the promoters of *HSP26* and *HSP30* are sufficient to increase protein synthesis during glucose starvation, despite repression of bulk translation activity (Ashe et al., 2000). Therefore, to examine whether the *ADH7* promoter enables protein synthesis under severe vanillin stress, we fused the *ADH7* promoter and terminator regions to the ORFs of other genes, including *GFP* and *GPX2*. Cells carrying pRS316-*ADH7*_Pro_-*GFP-ADH7*_Ter_ showed a clear GFP signal after treatment with vanillin, although the signal was negligibly detected under non-stressed conditions (**Figure 5A**). Likewise, increased protein levels of GFP and Gpx2 were induced by severe vanillin stress in cells carrying pRS316-*ADH7*_Pro_-*GFP-ADH7*_Ter_ or pRS316-*ADH7*_Pro_-*GPX2-FLAG-ADH7*_Ter_, as shown by western blot analysis (**Figure 5B**). Although the mRNAs driven by the *ADH7* promoter could not be effectively translated in 1 h treatment with 15 mM vanillin (**Figures 5B** and **2B**), the protein levels were gradually increased by the prolonged treatment (**Figure 5C**). These results may suggest that the rate of translation was markedly decelerated by 15 mM vanillin but the *ADH7* promoter-driven mRNAs can be slowly but preferentially translated under the severe vanillin stress.

Additionally, we examined whether the terminator region of *ADH7* affects gene expression upon vanillin stress. Cells carrying pRS316-*ADH7*_Pro_-*GPX2-FLAG-ADH6*_Ter_ showed almost the same levels of Gpx2 protein as cells containing pRS316-*ADH7*_Pro_-*GPX2-FLAG-ADH7*_Ter_ (**Figure 5B**). Likewise, expression of *GFP* from pRS316-*ADH7*_Pro_-*GFP-ADH6*_Ter_-transfected cells was similar to that from pRS316-*ADH7*_Pro_-*GFP-ADH7*_Ter_-transfected cells. These results clearly demonstrate that the *ADH7* promoter region enables the expression of its regulated genes under severe vanillin stress and that the *ADH7* terminator region has little effect on the induction of gene expression upon vanillin stress.

**FIGURE 6:**
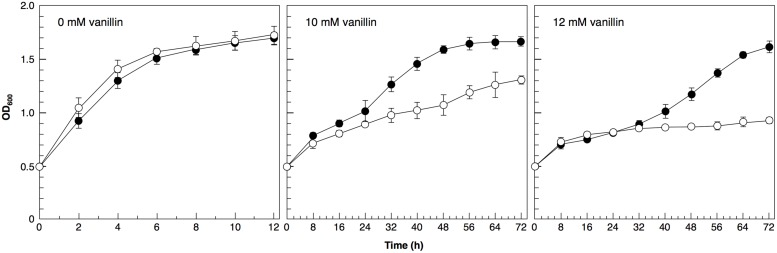
**Cells were cultured at 28°C in SD medium containing 10 or 12 mM vanillin.** Cell growth was monitored by measuring the optical density at 600 nm (OD_600_). Data are shown as the mean ± SD of three independent experiments. The strains examined were as follows: open circles, cells carrying pRS423; closed circles, cells carrying pRS423-*ADH7*_Pro_-*ADH6-ADH7*_Ter_.

Furthermore, we investigated the application of the *ADH7* promoter. We verified that expression of the *ADH6* gene under the control of the *ADH7* promoter in a multicopy vector (pRS423) improved the tolerance to severe vanillin stress (**Figure 6**). These results clearly indicate that the *ADH7* promoter is useful to improve the vanillin tolerance of yeast cells.

## Discussion

We demonstrated that vanillin caused transcriptional activation of both the *ADH6* and *ADH7* genes (**Figure 2A**). It has previously been shown that the expression of *ADH6* and *ADH7* can be activated by Yap1 and Msn2 (Berry and Gasch, 2008; Ma and Liu, 2010; Huebert et al., 2012), which, in turn, can be activated by vanillin (Nguyen et al., 2014a,b); therefore, it is likely that these transcription factors presumably contributed to the elevated mRNA levels of *ADH6* and *ADH7* observed upon vanillin stress.

We also demonstrated that the *ADH6* and *ADH7* genes exhibited different protein expression patterns in response to severe vanillin stress. While Adh6 protein levels were not increased in response to severe vanillin stress, Adh7 protein levels were significantly increased (**Figure 2B**). Similar to that observed with Adh6 protein, levels of Gpx2 and Trx2 proteins, encoded by Yap1-target genes *GPX2* and *TRX2*, could not be increased upon vanillin stress (data not shown). Because *GPX2*, *TRX2* (Nguyen et al., 2014a,b), and *ADH6* (**Figure 2A**) genes were transcriptionally activated by vanillin, the lack of increase in the protein expression of these genes might be caused by translational repression of mRNAs under conditions of severe vanillin stress. On the other hand, the inducible protein expression pattern of Adh7 clearly indicates that *ADH7* mRNA can be efficiently translated even under severe vanillin stress. Additionally, the expression pattern of *ADH7* mRNA was similar in *adh6*Δ cells and wild-type cells (**Figure 3**), indicating that the expression of *ADH7* is not a compensatory mechanism for a deficiency in Adh6.

**FIGURE 7:**
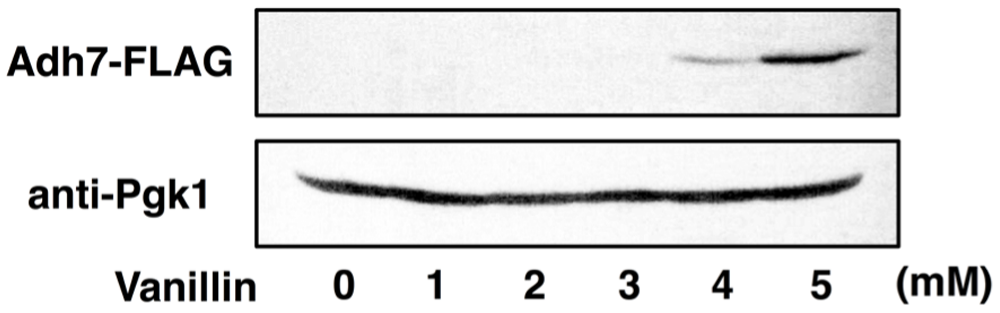
**Expression of the *ADH7* gene in the presence of low concentrations of vanillin.** Levels of Adh7 protein expression were determined by western blot analysis using an anti-FLAG antibody. Cells carrying a FLAG-tagged chromosomal copy of the *ADH7* gene were treated with vanillin. Pgk1 was used as a loading control. Cells were treated with the indicated concentrations of vanillin for 60 min.

Although Adh6 and Adh7 have similar functions and share high sequence identity (Larroy et al., 2002a), it has been reported that *adh7*Δ cells were able to reduce 2.5 mM vanillin more rapidly than *adh6*Δ cells could (Iwaki et al., 2013b), indicating that Adh6 plays a more dominant role than does Adh7 in the detoxification of low concentrations of vanillin. In this study, we examined the relative importance of Adh6 and Adh7 in the tolerance to severe vanillin stress. We found that *adh7*Δ cells reduced 8 mM vanillin less efficiently and grew more slowly than did *adh6*Δ cells. Because *ADH7* expression could not be induced by 2.5 mM vanillin (**Figure 7**) but was highly induced by 8 mM vanillin, these results suggest that Adh7 is of extreme importance when cells are challenged with high concentrations of vanillin. In addition, the protein levels of Adh6 gradually decreased, whereas Adh7 expression was induced and maintained at high levels after long-term cultivation with 8 mM vanillin (**Figure 4B**), suggesting that these proteins have different importance at different stages of response to vanillin stress, despite having similar functions. The constitutive expression of *ADH6* might be important in the initial response to the shock of vanillin stress, and yeast cells are presumably able to cope with relatively mild vanillin stress using Adh6. On the other hand, the drastic and stable induction of *ADH7*-expression seems to be more important for long-term tolerance to severe vanillin stress.

Zid and O’Shea (2014) reported that different promoter sequences affect translation of mRNAs under severe stress conditions. They found that the promoter region of genes containing heat shock elements (HSEs) enabled mRNAs to be translated during glucose starvation. We also demonstrated that the different promoters of the *ADH6* and *ADH7* genes were responsible for the different expression patterns upon severe vanillin stress. Replacement of ORFs and terminators did not affect the regulatory efficiency of *ADH6* and *ADH7* promoters under vanillin stress, and we were able to induce the expression of *GPX2* and *GFP* genes under severe vanillin stress using the *ADH7* promoter. It is conceivable that the *ADH7* promoter contains critical sequences that enable translation of mRNAs under severe vanillin stress. Intriguingly, an HSE (5′-TGAATTTTCG-3′) can be found in the *ADH7* promoter (from positions –473 to –464) but not in the *ADH6* promoter. The HSE in the promoter may contribute to efficient protein synthesis under severe vanillin stress as well as glucose starvation.

Because bulk translational activity was repressed in the presence of high concentrations of vanillin, it is apparent that most promoters cannot drive gene overexpression under this condition. The ability of the *ADH7* promoter to induce protein expression despite severe vanillin stress may be useful to improve the efficiency of bioethanol production. The expression of the *ADH6* gene driven by the *ADH7* promoter actually improved the growth of yeast cells under vanillin stress (**Figure 6**).

In addition to vanillin, lignocellulosic biomass hydrolysate usually contains a number of other fermentation inhibitors such as furaldehydes and acetic acid (Palmqvist and Hahn-Hägerdal, 2000b; Helle et al., 2003; Klinke et al., 2004; Graves et al., 2006; Shreaz et al., 2011; Vilela-Moura et al., 2011; Iwaki et al., 2013a). Therefore, overexpression of multiple genes involved in detoxification of these inhibitors as well as vanillin might be important to improve overall fermentation efficiency in biofuel production. However, overexpression of multiple genes using high-copy plasmids or constitutive strong promoters often generates intrinsic noise that affects cellular fitness and is generally harmful to yeast cells under non-stress conditions (Wang and Zhang, 2011). On the other hand, the vanillin-specific induction of gene expression caused by the *ADH7* promoter seems to provide a significant advantage for yeast well-being. Using the *ADH7* promoter to manipulate the gene expression would be an effective approach in the development of a robust industrial yeast strain for bioethanol fermentation from lignocellulosic hydrolysate.

## Author Contributions

SI did several experiments and mainly prepared the manuscript. TN did most of experiments and prepared the manuscript. AI did several important experiments.

## Conflict of Interest Statement

The authors declare that the research was conducted in the absence of any commercial or financial relationships that could be construed as a potential conflict of interest.
